# Comparison of human cytochrome P450 1A1-catalysed oxidation of benzo[*a*]pyrene in prokaryotic and eukaryotic expression systems

**DOI:** 10.1007/s00706-017-2002-0

**Published:** 2017-07-10

**Authors:** Marie Stiborová, Radek Indra, Michaela Moserová, Lucie Bořek-Dohalská, Petr Hodek, Eva Frei, Klaus Kopka, Heinz H. Schmeiser, Volker M. Arlt

**Affiliations:** 1 Department of Biochemistry, Faculty of Science, Charles University, Albertov 2030, 128 40 Prague 2, Czech Republic; 2 Division of Radiopharmaceutical Chemistry, German Cancer Research Center (DKFZ), Im Neuenheimer Feld 280, 69120 Heidelberg, Germany; 3 Analytical and Environmental Sciences Division, MRC-PHE Centre for Environment and Health, King’s College London, London, SE1 9NH UK; 4 NIHR Health Protection Research Unit in Health Impact of Environmental Hazards at King’s College London in Partnership with Public Health England, London, SE1 9NH UK

**Keywords:** DNA, Enzymes, Coenzymes, Membranes, Proteins

## Abstract

**Abstract:**

Cytochrome P450 (CYP) 1A1 is the most important enzyme activating and detoxifying the human carcinogen benzo[*a*]pyrene (BaP). In the previous studies, we had shown that not only the canonic NADPH:CYP oxidoreductase (POR) can act as electron donor but also cytochrome *b*_5_ and its reductase, NADH:cytochrome *b*_5_ reductase. Here, we studied the role of the expression system used on the metabolites generated and the levels of DNA adducts formed by activated BaP. We used an eukaryotic and a prokaryotic cellular system (Supersomes, microsomes isolated from insect cells, and Bactosomes, a membrane fraction of *Escherichia coli*, each transfected with *cDNA* of human CYP1A1 and POR). These were reconstituted with cytochrome *b*_5_ with and without NADH:cytochrome *b*_5_ reductase. We evaluated the effectiveness of each cofactor, NADPH and NADH, to mediate BaP metabolism. We found that both systems differ in catalysing the reactions activating and detoxifying BaP. Two BaP-derived DNA adducts were generated by the CYP1A1-Supersomes, both in the presence of NADPH and NADH, whereas NADPH but not NADH was able to support this reaction in the CYP1A1-Bactosomes. Seven BaP metabolites were found in Supersomes with NADPH or NADH, whereas NADPH but not NADH was able to generate five BaP metabolites in Bactosomes. Our study demonstrates different catalytic efficiencies of CYP1A1 expressed in prokaryotic and eukaryotic cells in BaP bioactivation indicating some limitations in the use of *E. coli* cells for such studies.

**Graphical abstract:**

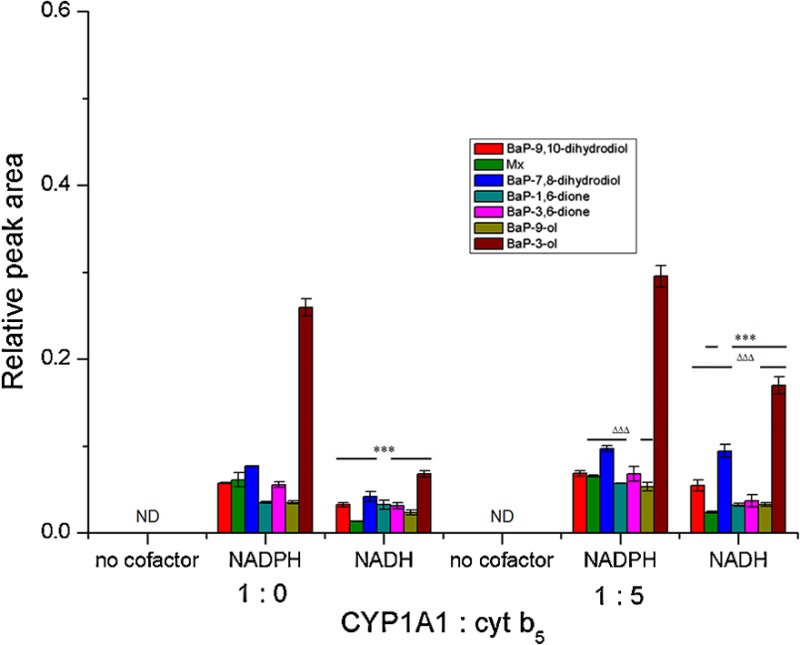

## Introduction

**Fig. 1:**
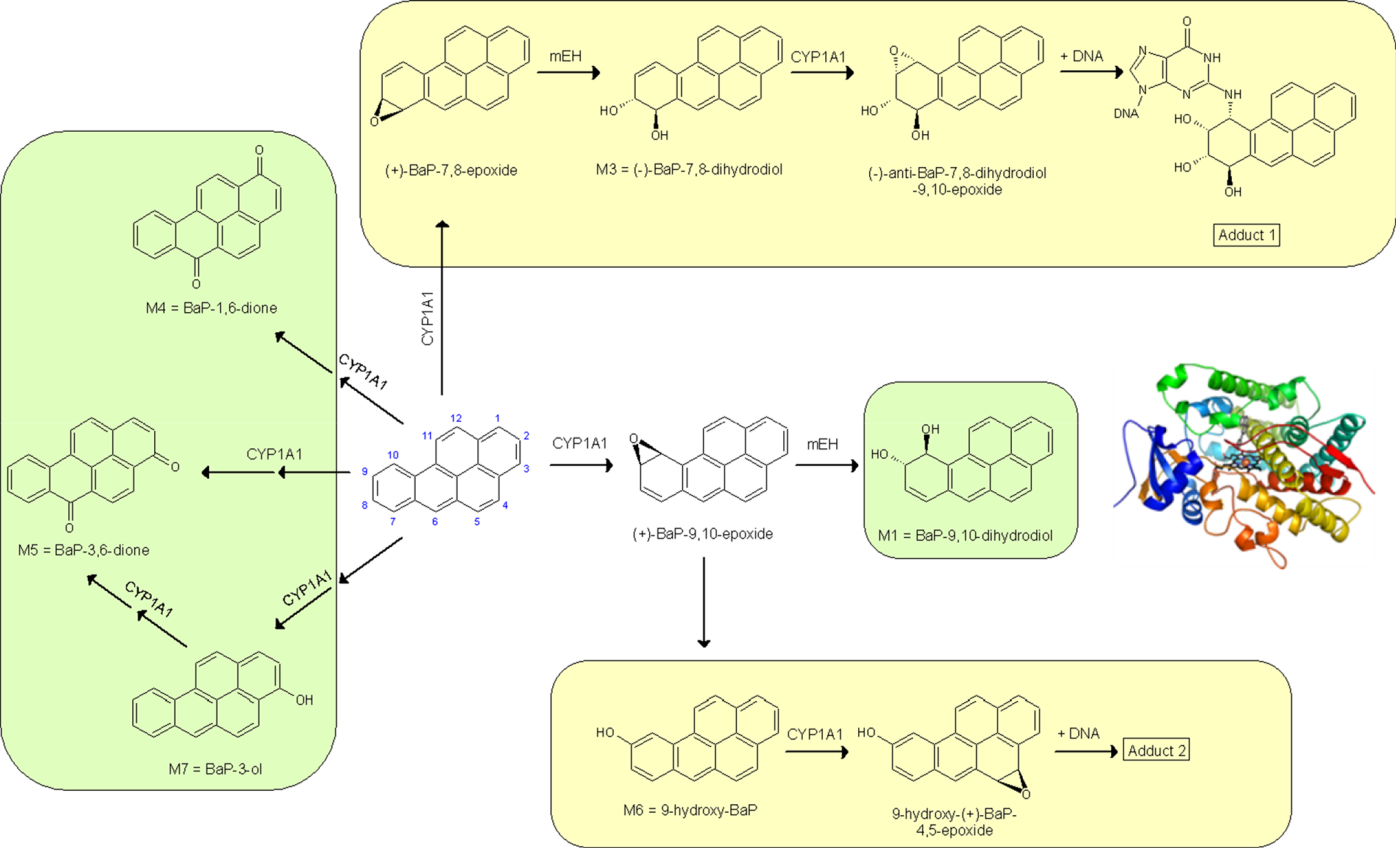
Proposed pathways of biotransformation and DNA adduct formation of BaP catalysed by CYP1A1 and EH. The typical three-step activation process by CYP1A1 followed by hydrolysis by EH leads to BPDE which forms dG-*N* ^2^-BPDE (adduct 1) and the two-step activation process by CYP1A1 leads to the formation of 9-hydroxy-BaP-4,5-epoxide that can react with deoxyguanosine in DNA (adduct 2) are shown in *yellow*. Formation of detoxification metabolites is shown in *green*. *Insert*: Structure of human CYP1A1 protein in a complex with α-naphthoflavone [12]

Benzo[*a*]pyrene (BaP) (Fig. 1) is a polycyclic aromatic hydrocarbon (PAH), which has been classified as human carcinogen (Group 1) by the International Agency for Research on Cancer [1]. In addition to the amount ingested, the activation of BaP is crucial for its carcinogenic potential [2]. BaP activation is catalysed by cytochrome P450 (CYP) enzymes, but it is also detoxified by these enzymes to metabolites that are excreted [3]. CYP1A1 was found to be the CYP primarily involved in the metabolic activation of BaP to species forming DNA adducts [3–6]. CYP1A1 oxidises BaP to an epoxide that is then converted to BaP-7,8-dihydrodiol by microsomal epoxide hydrolase (EH). Further bioactivation by CYP1A1 leads to the ultimate reactive species, BaP-7,8-dihydrodiol-9,10-epoxide (BPDE) that can react with DNA, forming adducts preferentially at guanine residues (Fig. 1). The 10-(deoxyguanosin-*N* ^2^-yl)-7,8,9-trihydroxy-7,8,9,10-tetrahydro-BaP (dG-*N* ^2^-BPDE) adduct (Fig. 1) is the major product of the reaction of BPDE with DNA in vitro and in vivo [4, 5, 7–11].

CYP1A1, however, is also responsible for BaP detoxification; BaP-dihydrodiols, BaP-diones, and hydroxylated BaP, some of which are excreted [3, 9, 13–16]. One of these metabolites, 9-hydroxy-BaP (BaP-9-ol), is formed predominantly by CYP1A1 and is a precursor of 9-hydroxy-BaP-4,5-epoxide, which can also form an adduct with deoxyguanosine in DNA (Fig. 1) [4, 5, 10, 11, 17–19]. Many other CYP enzymes, CYP1B1, 2B6, 2C8, 2C9, 2C19, and 3A4, have all been reported to oxidise BaP, but their efficiencies are about one order of magnitude lower than CYP1A1 [3, 9, 14, 20]. However, several studies have reported controversial results on the potency of CYP1A1 as well as other CYP enzymes to oxidise BaP [3, 6, 9, 14, 15, 21–30]. The reasons for these discrepancies are still a matter of debate and remain to be explored.

**Fig. 2:**
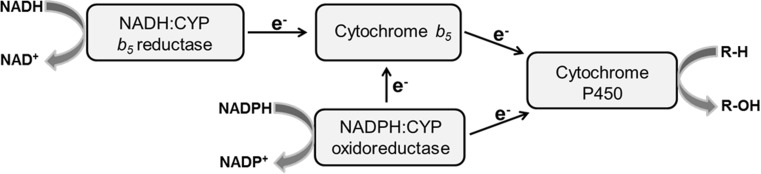
Electon transport pathways to CYPs in the endoplasmatic reticulum (adopted from [32])

CYP enzymes are a component of the monooxygenase system located in the membrane of the endoplasmic reticulum (microsomes). CYPs function by catalysing the insertion of one atom of molecular oxygen into a variety of xenobiotics, including BaP, while reducing the other oxygen atom to water, a reaction that requires two electrons [31]. These are classically supplied by NADPH:CYP oxidoreductase (POR) [31]. Early studies with reconstituted rat CYPs had, however, indicated a role for cytochrome *b*_5_ in NADH-dependent hydroxylation of BaP [21, 22]. We and others have since shown that the second electron needed for the reduction of CYPs may also be provided by cytochrome *b*_5_ which may be reduced by POR and NADPH or by NADH:cytochrome *b*_5_ reductase (Fig. 2) [28, 31–36]. Moreover, recently, we have demonstrated that the NADH:cytochrome *b*_5_ reductase/cytochrome *b*_5_ system can even act as sole electron donor for both reduction steps in the CYP1A1-reaction cycle catalysing the oxidation of BaP [4, 5]. Therefore, the levels of individual enzyme components and amounts relative to the CYP1A1 monooxygenase systems can dramatically influence CYP efficiencies to oxidise BaP.

Several systems containing CYP, such as hepatocytes, hepatic microsomes, purified CYP enzymes reconstituted with POR or NADH:cytochrome *b*_5_ reductase and cytochrome *b*_5_ with or without EH in liposomes, and/or recombinant CYP enzymes expressed in various cellular expression systems, have been utilised to study the metabolism of xenobiotics, including BaP in vitro (see [4, 5, 19–30, 36–41]). For reasons not yet unravelled, the yield in BaP metabolites and their pattern vary on the system studied. The present study was performed to follow up our previous work, where we showed that the NADH:cytochrome *b*_5_ reductase/cytochrome *b*_5_ system [4, 5] can act as sole electron donor in CYP1A1 catalysed BaP oxidation in Supersomes. In those studies, the results in liposomes reconstituted with isolated CYP1A1, NADH:cytochrome *b*_5_ reductase/cytochrome *b*_5_ with or without POR were, however, not the same as in Supersomes, which express CYP1A1 and POR and were reconstituted with cytochrome *b*_5_. In the present study, we used a third defined in vitro system, namely, a membrane fraction isolated from *Escherichia coli* transfected with human CYP1A1 and POR (Bactosomes). These were reconstituted with NADH:cytochrome *b*_5_ reductase/cytochrome *b*_5_ system. The role of NADH:cytochrome *b*_5_ reductase/cytochrome *b*_5_ in BaP metabolism by CYP1A1 in these distinct environments was studied.

## Results and discussion

### Oxidation of BaP by human CYP1A1 expressed in Supersomes and Bactosomes in the presence of NADPH or NADH

We compared the oxidation of BaP by two enzymatic systems containing human recombinant CYP1A1, namely, eukaryotic Supersomes and prokaryotic Bactosomes. Because Supersomes are microsomes (particles of broken endoplasmic reticulum), other enzymes of the endoplasmic reticulum membrane (i.e., NADH:cytochrome *b*_5_ reductase, EH, and cytochrome *b*_5_) are also expressed at basal levels as declared by the supplier (Gentest Corp., Woburn, MI, USA) [4]. The second experimental system we used was Bactosomes the membrane fraction of *E. coli* (prokaryotic) cells, in which human CYP1A1 and POR are over-expressed (Cypex, BioDundee, Dundee, UK). This system has been found to catalyse the CYP-mediated metabolism of various xenobiotics [42–45].

**Fig. 3:**
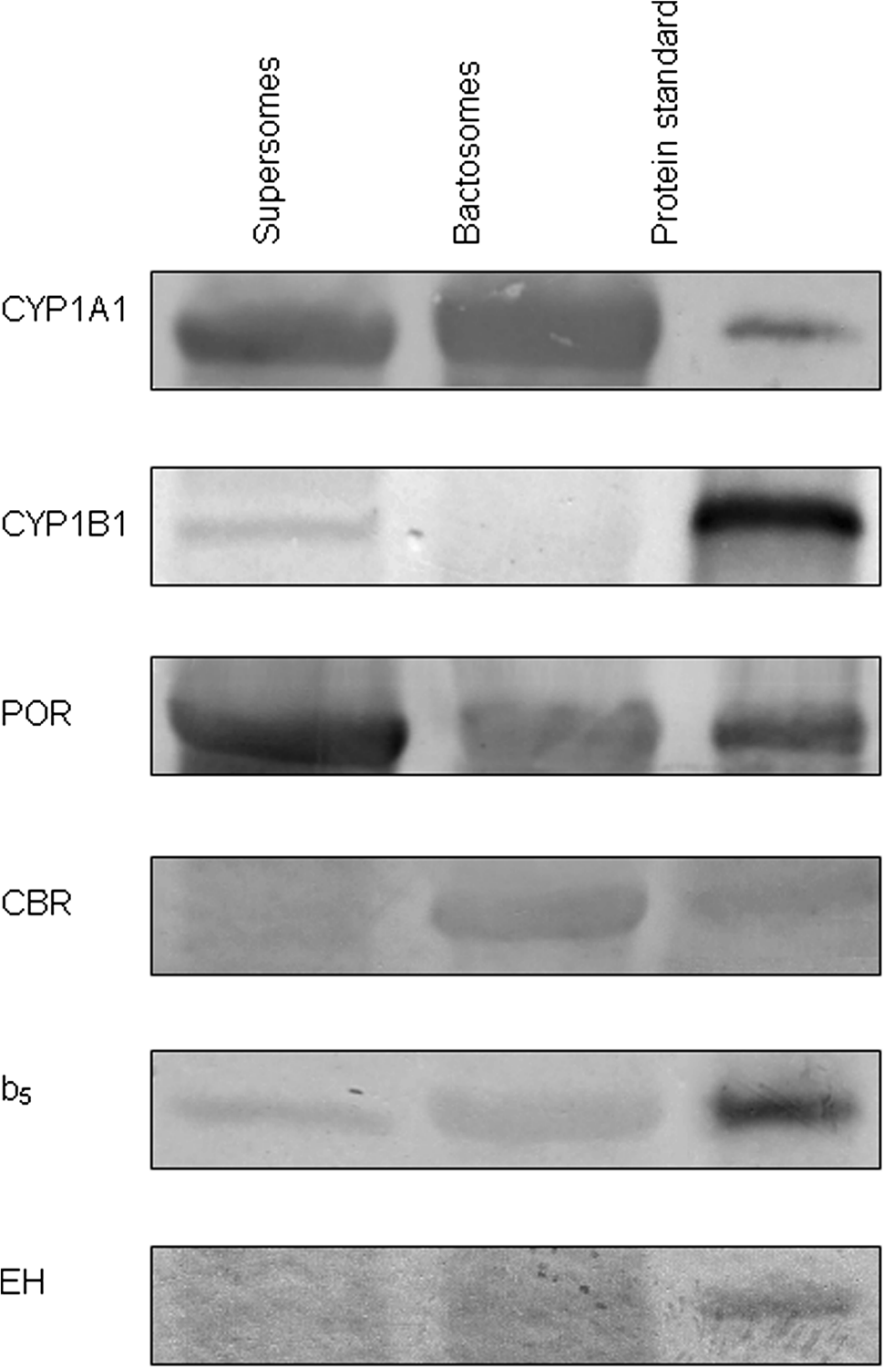
Western blot analysis of CYP1A1, CYP1B1, POR, NADH:cytochrome *b*_5_ reductase (CBR) cytochrome *b*_5_ (b_5_), and epoxide hydrolase (EH) in CYP1A1-Supersomes and CYP1A1-Bactosomes. For Western blotting, 25 μg of protein was used for the analysis of CYP1A1, CYP1B1, POR, CBR, and b_5_ and 50 μg of protein for the analysis of EH. Representative blots are shown; analyses were performed at least in duplicate

To confirm the presence of enzymes essential for the conversion of BaP in each CYP1A1 system, their expression was analysed by Western blotting using antibodies against the mammalian proteins (Fig. 3).

Figure 3 shows that similar levels of human CYP1A1 were over-expressed in Supersomes and Bactosomes, while the amount of POR was higher in Supersomes than in Bactosomes. Because BaP is also a substrate for CYP1B1 [14, 20], its expression was also analysed. Interestingly, employing the anti-human CYP1B1 antibody, CYP1B1 was detectable in Supersomes at basal levels (in amounts much lower than CYP1A1). In contrast, its expression was essentially not detectable in Bactosomes, as expected, since these are prokaryotic membranes. Cytochrome *b*_5_ and its reductase, NADH:cytochrome *b*_5_ reductase, were detectable in both systems, but levels were much lower than those of POR. Expression levels of EH were close to the detection limit of the Western blot method.

**Fig. 4:**
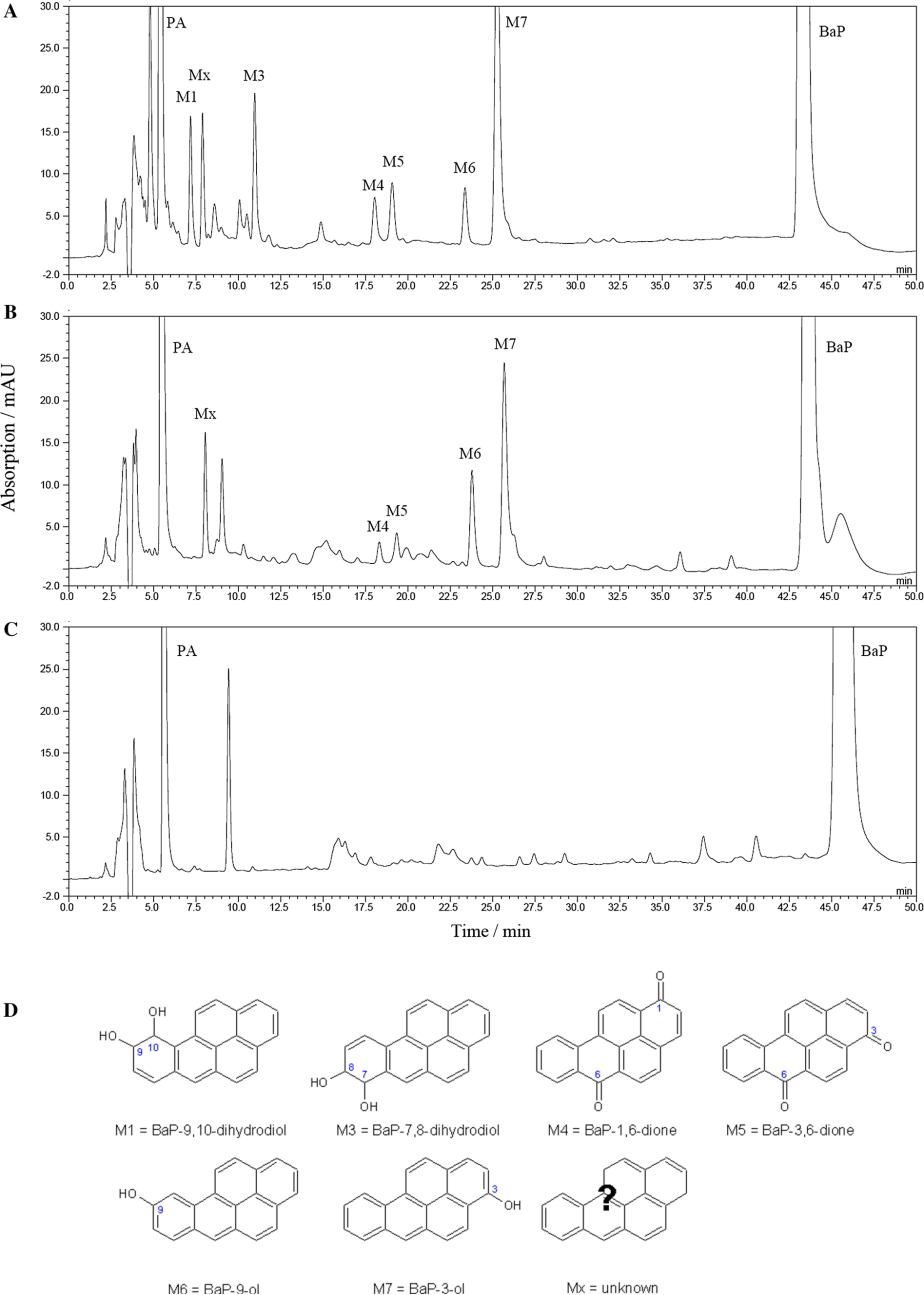
HPLC chromatograms of BaP metabolites formed by human recombinant CYP1A1 expressed in Supersomes (**a**) [4] and human recombinant CYP1A1 expressed in a membrane of *E. coli* (Bactosomes) (**b**) in the presence of NADPH. **c** Control incubation mixture containing BaP and bactosomes but no NADPH. **d** Structures of BaP metabolites (M1, M3–M7, and Mx). *PA* phenacetin

To examine whether indeed cytochrome *b*_5_ can act as sole electron donor in CYP1A1-catalysed BaP oxidation in both membrane systems, Supersomes and Bactosomes were reconstituted with isolated cytochrome *b*_5_ at a molar ratio of CYP1A1:cytochrome *b*_5_ of 1:5. NADPH or NADH, cofactors of POR, and NADH:cytochrome *b*_5_ reductase, respectively, were utilised to examine CYP1A1-mediated BaP oxidation. BaP metabolites formed by human CYP1A1 in these enzyme systems were analysed by HPLC (Fig. 4).

**Fig. 5:**
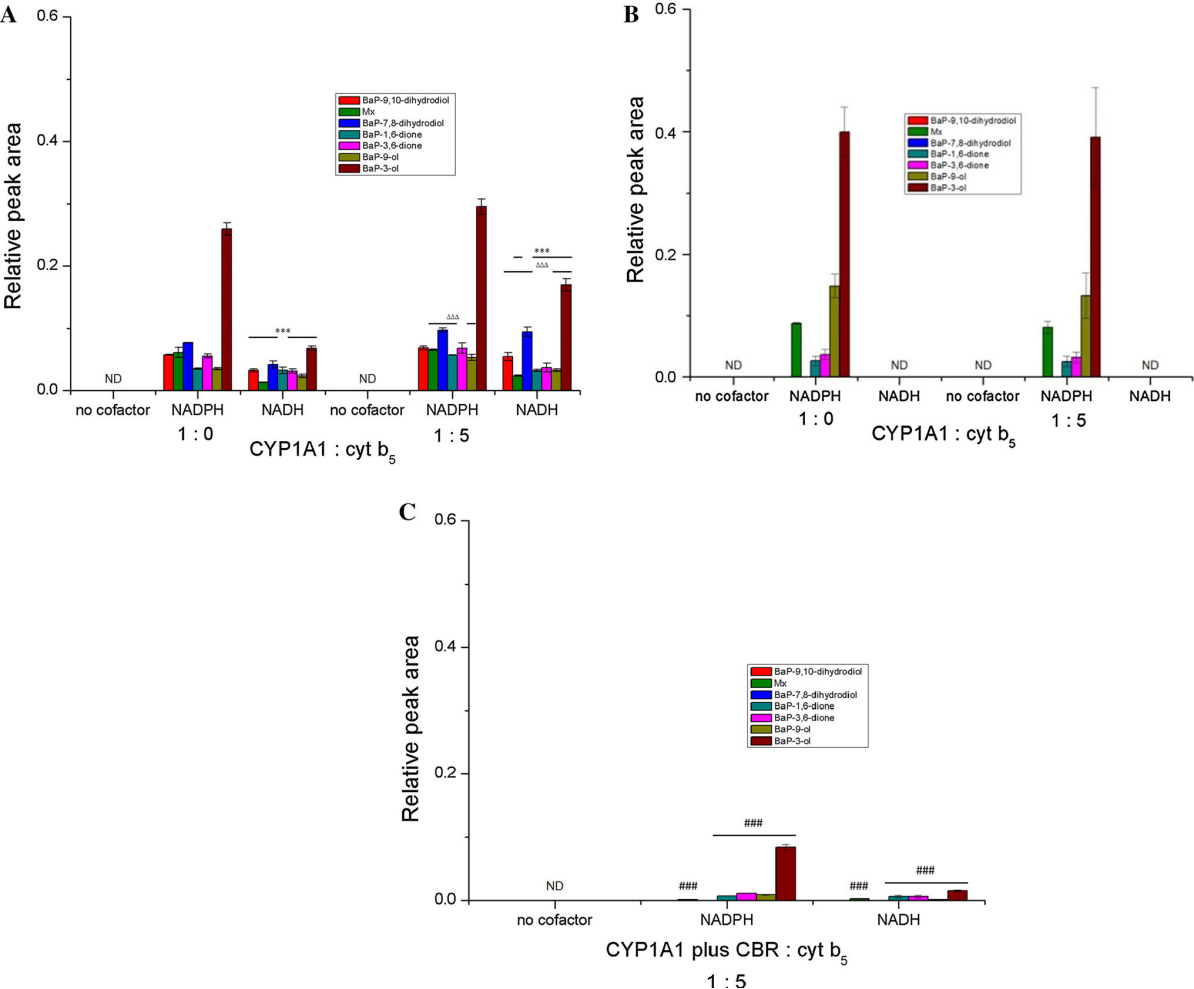
Metabolism of BaP by human recombinant CYP1A1 in Supersomes (**a**) [4] and Bactosomes **b**, **c** in the presence of either NADPH or NADH and the effect of cytochrome *b*_5_ (at a molar ratio of CYP1A1:cytochrome *b*_5_ of 1:5) (**a**, **b**) or NADH:cytochrome *b*_5_ reductase (CBR) (at a molar ratio of CYP1A1:CBR of 1:1) (**c**) on this metabolism. BaP metabolites were measured by HPLC analysis. Supersomes and Bactosomes containing basal levels of NADH:cytochrome *b*_5_ reductase and cytochrome *b*_5_ were reconstituted with additional cytochrome *b*_5_ (at a molar ratio of CYP1A1:cytochrome *b*_5_ of 1:5). Bactosomes shown in (C) were reconstituted with additional NADH:cytochrome *b*_5_ reductase (CBR) and cytochrome *b*_5_ (CYP1A1 plus CBR plus cyt b_5_) at a molar ratio of CYP1A1:CBR:cyt b_5_ of 1:1:5). Values represent mean ± SD from three parallel measurements. *ND* not detected. ***P < 0.001 (Student’s *t* test), significantly different from incubations using NADPH as cofactor (**a**); ^ΔΔΔ^ *P* < 0.001 (Student’s *t* test), significantly different from incubations without cytochrome *b*_5_ (**a**); ^###^ *P* < 0.001 (Student’s *t* test), significantly different from incubations in Bactosomes with NADPH or NADH without CBR (**c**)

We show here results from our previous work [4] with Supersomes (Figs. 4a, 5a) to be able to compare them to our new results in Bactosomes. Seven BaP metabolites were generated by human CYP1A1 in Supersomes both in the presence of NADPH and NADH (Figs. 4a, 5a). They were structurally identified previously [19, 20, 29] as BaP-9,10-dihydrodiol (M1), BaP-7,8-dihydrodiol (M3), BaP-1,6-dione (M4), BaP-3,6-dione (M5), BaP-9-ol (M6), and BaP-3-ol (M7) (for structures, see Fig. 4d). In addition, a metabolite of unknown structure (Mx) was detected. BaP-4,5-dihydrodiol (M2), which is a BaP metabolite generated by rat CYP1A1 [19, 28], has not been detected in the Supersome system with human CYP1A1. The metabolites found in this CYP1A1 system indicated that BaP is metabolised not only by CYP1A1 but also by EH, which is important for the hydration of BaP-epoxides to produce dihydrodiols. NADH was less effective than NADPH as electron donor to human CYP1A1 in Supersomes (Fig. 5a). The addition of cytochrome *b*_5_ to the incubation mixtures with Supersomes at a molar ratio of CYP1A1 to cytochrome *b*_5_ of 1:5 led to an increase in CYP1A1-mediated BaP oxidation both in the presence of NADPH and NADH (Fig. 5a). The highest stimulation effect of cytochrome *b*_5_ was seen on the generation of BaP-7,8-dihydrodiol in the presence of NADPH or NADH as well as on the formation of BaP-9,10-dihydrodiol, metabolite Mx, and BaP-3-ol in the presence of NADH (Fig. 5a). No BaP metabolites were found when NADPH or NADH was omitted from the incubation mixtures containing CYP1A1-Supersomes (Fig. 5a).

In the presence of NADPH, human recombinant CYP1A1 expressed in Bactosomes was also capable of oxidising BaP. However, only five BaP metabolites were detectable in this system, namely, BaP-1,6-dione, BaP-3,6-dione, BaP-9-ol, BaP-3-ol, and metabolite Mx, whereas no BaP-dihydrodiols were detected (Figs. 4b, 5b). The same BaP metabolites have been found previously in experiments employing pure human recombinant CYP1A1 reconstituted with POR without any other enzymes (i.e., EH) in liposomes [4]. These results indicated that EH, which catalyses the hydration of BaP-epoxides to dihydrodiols, even though expressed at low levels in Bactosomes, is unable to produce BaP-dihydrodiols in amounts detectable by HPLC. Except for BaP-1,6-dione and BaP-3,6-dione, which were produced by the enzymes in the prokaryotic system at levels similar to those formed by CYP1A1 in Supersomes, the three other metabolites, BaP-3-ol, BaP-9-ol, and metabolite Mx, were formed in Bactosomes in significantly higher amounts than by the CYP1A1-Supersome system (*P* < 0.001 for BaP-9-ol and BaP-3-ol and *P* < 0.05 for Mx) (Fig. 5). 3.5-Fold higher amounts of BaP-9-ol were formed in Bactosomes than Supersomes, even though POR is expressed in Bactosomes in lower levels than in Supersomes. Likewise, these three BaP metabolites were formed more efficiently than BaP-1,6-dione and BaP-3,6-dione in liposomes with CYP1A1 reconstituted with POR used in our previous work [4]. No BaP metabolites were found when NADPH was omitted from the incubation mixtures containing CYP1A1-Bactosomes (Figs. 4c, 5b).

NADH was ineffective as cofactor for BaP oxidation in the CYP1A1-Bactosome system. This finding confirms that NADH is a very poor coenzyme of POR over-expressed in Bactosomes, leading to metabolite levels that are negligible relative to NADPH. Recently, we found the same results using cytochrome *c* as a substrate for POR [4, 5]. In contrast to the stimulating effect of cytochrome *b*_5_ on BaP oxidation by human CYP1A1 in Supersomes, no such effect was detected in the CYP1A1 system expressed in *E. coli* (Fig. 5b). Even in the presence of the basal level of NADH/cytochrome *b*_5_ reductase (Fig. 3) plus cytochrome *b*_5_ with which the CYP1A1 in Bactosomes was reconstituted at a ratio of 1:5 (Fig. 5b), no effect of NADH was detectable. However, after the reconstitution of Bactosomes with NADH:cytochrome *b*_5_ reductase and cytochrome *b*_5_ (at a ratio of CYP1A1:NADH:cytochrome *b*_5_ reductase:cytochrome *b*_5_ of 1:1:5), these oxidized BaP in the presence of NADH (Fig. 5c). These results confirmed the previous findings that the NADH/cytochrome *b*_5_ reductase/cytochrome *b*_5_ system can function as a sole electron donor for CYP1A1 in its catalytic cycle, acting independently of NADPH and POR [4, 5]. Nevertheless, the levels of BaP metabolites generated in CYP1A1-Bactosomes reconstituted with NADH:cytochrome *b*_5_ reductase and cytochrome *b*_5_ were much lower than their levels generated by human CYP1A1 reconstituted with these enzymes in liposomes [4].

Differences in membrane composition of the used CYP1A1 systems, therefore, seem to play an essential role in enzyme activity. This is described in a review by Schneiter and Toulmay [46] who emphasise that the lipid composition not only determines the sorting, orientation, and assembly into oligomeric complexes of integral membrane proteins but as a consequence also affects the activity of membrane proteins. This is particularly true for heterologous expression systems as in our case, where both systems are heterologous; however, CYP1A1 and POR expressed in the Supersomes seem to mimic the mammalian system more truly than the Bactosome system. The pattern of BaP metabolites is shifted in the latter system and the overall yield is not smaller but at least comparable to Supersomes. In addition to the expressed enzymes, the lipids of the membranes probably define functional uptake of added membrane proteins in the reconstitution experiments. In a highly artificial environment with liposomes consisting of dilauryl-phosphatidylcholine and CHAPS, cytochrome *b*_5_ also enhanced BaP oxidation by human CYP1A1 [4].

### Formation of BaP-DNA adducts by human CYP1A1 expressed in Supersomes and Bactosomes in the presence of NADPH or NADH

We further compared the formation of BaP-DNA adducts by human CYP1A1 expressed in Supersomes and Bactosomes using the ^32^P-postlabelling assay.

**Fig. 6:**
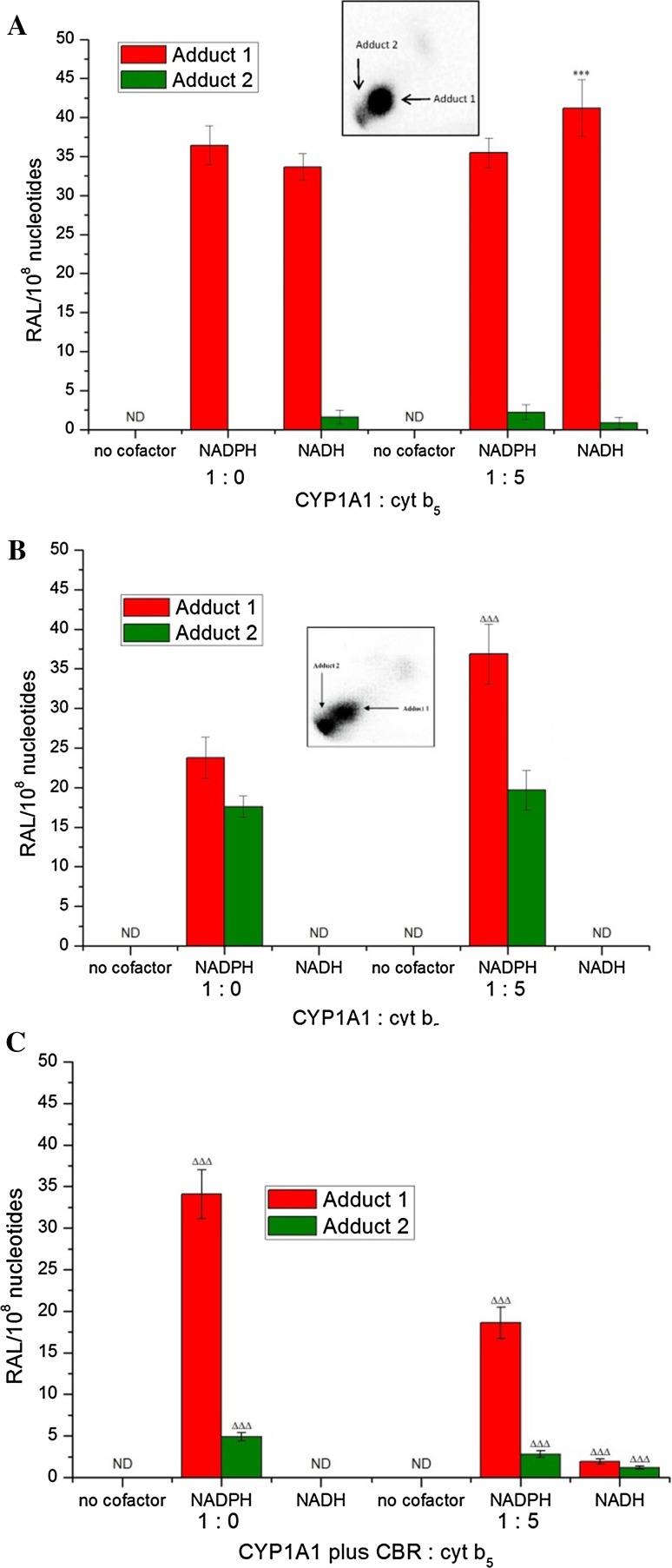
DNA adduct formation by BaP, measured by ^32^P-postlabelling, activated with human recombinant CYP1A1 in Supersomes (**a**) [4] and Bactosomes **b**, **c** in the presence of NADPH or NADH and the effect of cytochrome *b*_5_ (cyt b_5_, at a molar ratio of CYP1A1:cytochrome *b*_5_ of 1:5) on this reaction. Bactosomes shown in (**c**) were reconstituted with NADH:cytochrome *b*_5_ reductase (CBR) (CYP1A1 plus CBR) at a molar ratio of CYP1A1:CBR of 1:1. *Insert A and B*: Autoradiographic profiles of BaP-DNA adducts formed by human CYP1A1 in Supersomes and in Bactosomes in the presence of NADPH and cytochrome *b*_5_, respectively, as evaluated by thin-layer chromatography ^32^P-postlabelling as described previously [19]. Values represent mean total RAL (relative adduct labelling) ± SD (*n* = 3; analyses of three independent in vitro incubations). ND, not detected. ***P < 0.001 (Student’s *t* test), levels of BaP-adduct 1 formed by incubations with CYP1A1 in Supersomes significantly different from incubations with this enzymatic system without cytochrome *b*_5_ (A); ^ΔΔΔ^ *P* < 0.001 (Student’s *t* test), levels of BaP-adducts 1 and 2 formed by incubations with CYP1A1 in Bactosomes significantly different from incubations with this CYP1A1-Bactosomes system without CBR and cytochrome *b*_5_ (**b**, **c**)

In our previous work up, two DNA adducts (assigned adducts 1 and 2; see inserts in Fig. 6) were detected by ^32^P-postlabelling when BaP was activated with human CYP1A1 expressed in Supersomes in the presence of either NADPH or NADH [4]. In this system, BaP-DNA adduct 1 was predominant (Fig. 6a) [4]. Comparison with the previous ^32^P-postlabelling analyses [19, 47] showed that adduct 1 is the dG-*N* ^2^-BPDE adduct. The other adduct, a very minor DNA adduct in Supersomes, has similar chromatographic properties on thin-layer chromatography to a guanine adduct derived from the reaction with 9-hydroxy-BaP-4,5-epoxide (see adduct spot 2 in insert of Fig. 6). The structure of this adduct has not yet been identified. No such BaP-DNA adducts were found when NADPH or NADH was omitted from the incubation mixtures (Fig. 6a).

Surprisingly, in the presence of NADPH, human recombinant CYP1A1 over-expressed with POR in *E. coli* also generated BaP-DNA adducts 1 and 2 (Fig. 6), even though BaP-7,8-dihydrodiol, the precursor of BPDE, was not detectable by HPLC (Fig. 5b). This finding indicates that EH expressed in Bactosomes even at very low levels is capable of catalysing the hydration of BaP-7,8-epoxide to BaP-7,8-dihydrodiol. It appears that the very low amounts of BaP-7,8-dihydrodiol, undetectable by HPLC, generated BPDE to form dG-*N* ^2^-BPDE adducts detectable by the highly sensitive ^32^P-postlabelling method (Fig. 6). This suggestion is strongly supported by the previous results showing that the ^32^P-postlabelling method used to detect and quantify BaP-DNA adducts is up to six orders of magnitude more sensitive than HPLC [48]. Levels of the dG-*N* ^2^-BPDE adduct (adduct 1) formed by CYP1A1 in Bactosomes in the presence of NADPH were 1.6-fold lower than those in Supersomes in the presence of this cofactor (Fig. 6). The formation of adduct 2 by CYP1A1 expressed in Bactosomes was much higher than those formed in the CYP1A1-Supersomes. These results corresponded to higher amounts of BaP-9-ol formed in the CYP1A1-Bactosome system than in CYP1A1-Supersomes (BaP-9-ol is the precursor of 9-hydroxy-BaP-4,5-epoxide generating adduct 2) (Figs. 5, 6).

Both cytochrome *b*_5_ and NADH:cytochrome *b*_5_ reductase enhanced formation of adduct 1 (i.e., dG-*N* ^2^-BPDE) in reconstituted systems containing Bactosomes and NADPH (Fig. 6b, c). When NADH:cytochrome *b*_5_ reductase (at a ratio of CYP1A1:NADH:cytochrome *b*_5_ reductase of 1:1) plus cytochrome *b*_5_ (at a ratio of CYP1A1:cytochrome *b*_5_ of 1:5) were added together, levels of both BaP-DNA adducts were lower than with only cytochrome *b*_5_ added to the Bactosomes or in CYP1A1 Bactosomes alone. It is possible that the higher amounts of protein in the incubation mixtures might scavenge the reactive BaP metabolites, thereby decreasing BaP-DNA adduct formation. No BaP-DNA adducts were found when NADPH was omitted from the incubation mixtures containing human recombinant CYP1A1 in Bactosomes (Fig. 6b, c).

NADH was ineffective as cofactor for BaP activation by CYP1A1-Bactosomes (Fig. 6b, c), which again indicates that NADH:cytochrome *b*_5_ reductase and/or its substrate cytochrome *b*_5_ are not expressed in the membrane of *E. coli* in states appropriate for catalysis. Furthermore, these findings again indicate that NADH is a poor coenzyme of POR, over-expressed in Bactosomes. Only after the reconstitution of Bactosomes with NADH:cytochrome *b*_5_ reductase (at a ratio of CYP1A1: NADH:cytochrome *b*_5_ reductase of 1:1) plus cytochrome *b*_5_ (at a ratio of CYP1A1:cytochrome *b*_5_ of 1:5), these were able to mediate the formation of both BaP-DNA adducts in the presence of NADH (Fig. 6c). These results again confirmed the previous findings that the NADH/cytochrome *b*_5_ reductase/cytochrome *b*_5_ system can act as an exclusive electron donor for CYP1A1 in its catalytic cycle, functioning independently of NADPH and POR [4, 5]. Nevertheless, the levels of BaP-DNA adducts formed in Bactosomes reconstituted with NADH:cytochrome *b*_5_ reductase and cytochrome *b*_5_ were much lower than their levels generated by human CYP1A1 reconstituted with these enzymes in liposomes [4]. Here, again, the lipid composition affects the yield in DNA adducts.

## Conclusions

This study comparing two model systems containing human recombinant CYP1A1 either expressed in eukaryotic or prokaryotic cells (Supersomes or Bactosomes) demonstrates different efficiencies of these systems in BaP oxidation and the formation of BaP-derived DNA adducts. Even though the human CYP1A1 enzyme essential for BaP metabolism is over-expressed in both systems at similar levels, its effectiveness in BaP oxidation differed significantly. Our results strongly suggest that the lipid composition of the membranes of these subcellular systems is important for the yield of BaP metabolites and their pattern. This might be an explanation for the discrepancies in BaP metabolism observed by us and others from native systems such as hepatocytes to heterologous expression systems. This work also confirmed that expression systems using eukaryotic cells such as Supersomes most closely resemble the situation in human hepatic microsomes [4, 29]. All BaP metabolites formed by Supersomes were also formed by CYP1A1 in human hepatic microsomes and human bronchoalveolar H358 cells (expressing CYP1A1) after BaP exposure [15]. These results demonstrate that NADH:cytochrome *b*_5_ reductase, cytochrome *b*_5,_ and epoxide hydrolase were necessary to transform BaP to these metabolites, although expressed at low levels in the “supersomal” microsomes seems to be in functional states and as active as in human cells.

## Experimental

### Chemicals and CYP1A1 subcellular systems

BaP (CAS no. 50-32-8; purity ≥96%), NADH (as disodium salt; purity ~95%), and NADPH (as tetrasodium salt; ~98% purity) were obtained from Sigma Chemical Co (St Louis, MO, USA). CYP1A1-Supersomes, microsomes isolated from insect cells transfected with a baculovirus construct containing *c*DNA of human CYP1A1 and POR that are therefore over-expressed in these microsomes, were purchased from Gentest Corp. (Woburn, MI, USA). However, because they are microsomes, other enzymes (proteins) of the endoplasmic reticulum membrane (i.e., NADH:cytochrome *b*_5_ reductase, EH, and cytochrome *b*_5_) are also expressed at basal levels in these Supersomes™ (Gentest Corp., Woburn, MI, USA). Bactosomes, a membrane fraction isolated from cells of *E. coli* transfected with construct of *c*DNA of human *CYP1A1* and human *POR* and, therefore, over-expressed in these Bactosomes, were obtained from Cypex (BioDundee, Dundee, UK). Supersomes and Bactosomes were isolated from insect and *E. coli* cells, respectively, that were not transfected with NADH:cytochrome *b*_5_ reductase or cytochrome *b*_5_.

### Western Blot analysis

For the detection of CYP1A1, 1B1, EH, POR, NADH:cytochrome *b*_5_ reductase, and cytochrome *b*_5_ in CYP1A1-Supersomes and CYP1A1-Bactosomes, 25 μg or 50 μg (for EH) of protein were subjected to sodium dodecylsulfate polyacrylamide gel electrophoresis (SDS-PAGE) (4–20% Mini-PROTEAN^®^ TGX™ Gel 15 well, Bio-Rad) and applied onto a polyvinylidene fluoride (PVDF) membrane (Trans-Blot^®^ Turbo™ Mini PVDF Transfer Packs, Bio-Rad) as reported [11, 49]. The membrane was blocked in 5% (w/v) non-fat milk dissolved in PBS-Triton X-100 buffer [0.134 M NaCl, 1.8 mM Na_2_HPO_4_, 1 mM NaH_2_PO_4_; pH 7.2; 0.3% (w/v) Triton X-100] for 1 h at ambient temperature, then probed overnight at 4 °C with the following antibodies: human CYP1A1 was detected with a goat anti-rat CYP1A1 antibody (1:1250, Antibodies-online GmbH, Aachen, Germany), CYP1B1 with rabbit-anti-human CYP1B1 polyclonal antibody (G-25) (1:200, Santa Cruz Biotechnology, Dallas, Texas, USA), POR with rabbit-anti-human cytochrome P450 reductase polyclonal antibody (1:500, AbCam, MA, USA), NADH:cytochrome *b*_5_ reductase with goat anti-rat cytochrome *b*_5_ reductase 3 antibody (CYB5R3, C-Term; 1:1000, Antibodies-online GmbH, Aachen, Germany), EH with rabbit anti-human epoxide hydrolase polyclonal antibody (1:1000, AbCam, MA, USA), and cytochrome *b*_5_ with a rabbit anti-human cytochrome *b*_5_ polyclonal antibody (1:750, AbCam, MA, USA) diluted in 5% non-fat milk in PBS-buffered saline with Triton X-100 (PBS-Triton buffer). After washing in PBS-Triton, the antigen–antibody complexes were visualised with either alkaline phosphatase-conjugated goat anti-rabbit IgG antibody (1:1428, Sigma-Aldrich, USA) or chicken anti-goat IgG antibody (1:500, Santa Cruz Biotechnology, Dallas, Texas, USA), and 5-bromo-4-chloro-3-indolylphosphate/nitrobluetetrazolium (BCIP/NBT Color Development Substrate, Promega WI, USA) was used as chromogenic substrate. Antibody against glyceraldehyde phosphate dehydrogenase (GAPDH) (1:750, Millipore, Massachusetts, USA) was used as loading control as recommended by the manufacturer (not shown).

### Isolation of Rat NADH:cytochrome b_5_ reductase and rabbit cytochrome b_5_

Cytochrome *b*_5_ reductase (E.C. 1.6.2.2) was isolated from rat liver microsomes by a procedure described by Perkins and Duncan [51]. The specific activity of rat cytochrome *b*_5_ reductase measured as NADH-ferricyanide reductase was 49.2 μmol ferricyanide/min/mg protein. Cytochrome *b*_5_ was isolated from rabbit liver microsomes as described [52]. Both proteins purified to apparent homogeneity [51, 52] were utilised in the reconstitution experiments with Bactosomes (cytochrome *b*_5_ and NADH:cytochrome *b*_5_ reductase) and Supersomes (cytochrome *b*_5_).

### Incubations to study metabolism of BaP by human recombinant CYP1A1 in Supersomes or Bactosomes

Incubation mixtures used for studying BaP metabolism by Supersomes or Bactosomes contained in a final volume of 0.25 cm^3^ 100 mmol dm^−3^ potassium phosphate buffer (pH 7.4), 1 mmol dm^−3^ NADPH or NADH, 50 µmol dm^−3^ BaP (dissolved in 0.025 cm^3^ DMSO). With this DMSO concentration (1% as a final concentration), no inhibition of the NADPH-dependent CYP-catalysed oxidation of several substrates has been found previously [4–6, 9, 10, 19, 28, 29, 35] and 100 nmol dm^−3^ human recombinant CYP1A1 present with its reductase, POR, in Supersomes or Bactosomes. Control incubations contained no NADPH. The same amount of a solvent (DMSO) was used in control incubations without BaP. The reaction was initiated by adding NADPH or NADH. In the experiments, where the effect of cytochrome *b*_5_ on BaP metabolism by human CYP1A1 in Supersomes or Bactosomes was investigated, 500 nmol dm^−3^ of pure cytochrome *b*_5_ protein was added to reach a molar ratio of CYP1A1:cytochrome *b*_5_ of 1:5 and reconstituted with Supersomes or Bactosomes (see Figs. 5, 6). CYP1A1 enzyme (100 nmol dm^−3^) reconstitution utilizing Supersomes or Bactosomes with purified cytochrome *b*_5_ (500 nmol dm^−3^) was performed as described elsewhere [4–6, 9, 10, 19, 20, 28, 29, 35, 36, 49, 50]. Negative controls lacked either CYP1A systems or cofactors. After incubation (37 °C, 20 min), 0.005 cm^3^ 1 mmol dm^−3^ phenacetin in methanol was added as an internal standard. BaP metabolism by microsomes has been shown to be linear up to 30 min of incubation [4, 5, 19, 20]. BaP metabolites were extracted twice with ethyl acetate (2 × 1 cm^3^), solvent evaporated to dryness, residues dissolved in 0.025 cm^3^ methanol, and BaP metabolites separated by HPLC as reported previously [4, 5, 19, 20]. BaP metabolite peaks were identified by HPLC by comparison with metabolite standards, whose structures were determined previously by NMR and/or mass spectrometry [19].

### Determination of BaP-DNA adduct formation by ^32^P-postlabelling

Incubation mixtures used to assess DNA adduct formation by BaP activated with all enzymatic systems containing human CYP1A1 consisted of 50 mmol dm^−3^ potassium phosphate buffer (pH 7.4), 1 mmol dm^−3^ NADPH or NADH, 100 nmol dm^−3^ human recombinant CYP1A1 in Supersomes and reconstituted with 500 nmol dm^−3^ cytochrome *b*_5_ (at a ratio of CYP1A1:cytochrome *b*_5_ of 1:5) or in Bactosomes and reconstituted with 100 nmol dm^−3^ NADH:cytochrome *b*_5_ reductase and 500 nmol dm^−3^ cytochrome *b*_5_ as indicated in the figures, 0.1 mmol dm^−3^ BaP (dissolved in 0.0075 cm^3^ DMSO), and 0.5 mg of calf thymus DNA in a final volume of 0.75 cm^3^ as described previously [4, 19]. The reaction was initiated by adding 0.1 mmol dm^−3^ BaP and incubations were carried out at 37 °C for 60 min. BaP-DNA adduct formation has been shown to be linear up to 90 min [10, 19]. Control incubations were carried out either without CYP1A1 in Supersomes and in Bactosomes, without NADPH (or NADH), without DNA, or without BaP. After the incubation, BaP and metabolites were extracted with ethyl acetate and DNA was isolated from the residual water phase by the standard phenol/chloroform extraction. DNA adduct formation was analysed using the nuclease P1 version of the ^32^P-postlabelling technique [4, 5, 10, 19]. Resolution of the adducts by thin-layer chromatography using polyethylenimine-cellulose plates (Macherey and Nagel, Düren, Germany) was carried out as described [4, 5, 10, 19, 48]. DNA adduct levels (RAL, relative adduct labelling) were calculated as described [54]. As mentioned above, since Bactosomes contain very low levels of NADH/cytochrome *b*_5_ reductase according to the provider, we added this enzyme to the reconstituted system with Bactosomes (100 nmol dm^−3^), at a ratio of CYP1A1:NADH:cytochrome *b*_5_ reductase of 1:1).

### Statistical analyses

Statistical analyses were carried out with Student’s *t* test (Unistat Ltd, Highgate, London N6 5UQ, ENGLAND UK). Means ± standard deviations of three parallel experiments are shown and *P* values <0.05 were considered significant.
